# A qualitative analysis of post-hoc interviews with multilevel participants of a randomized controlled trial of a community-based intervention

**DOI:** 10.1371/journal.pone.0303075

**Published:** 2024-05-09

**Authors:** Nathan Kohrman, Mohamad Rashid, Roxana Flores, Ciantel Blyler, Noel C. Barragan, Tony Kuo, Moira Inkelas, Steven Chen, Florian Rader, Susan Cheng, Christine Albert, Natalie A. Bello, Joseph Ebinger

**Affiliations:** 1 Keck School of Medicine, University of Southern California, Los Angeles, California, United States of America; 2 Department of Medicine, Cedars-Sinai Medical Center, Los Angeles, California, United States of America; 3 Los Angeles County Department of Public Health, Division of Chronic Disease and Injury Prevention, Los Angeles, California, United States of America; 4 Department of Cardiology, Smidt Heart Institute, Cedars-Sinai Medical Center, Los Angeles, California, United States of America; 5 Department of Epidemiology, Los Angeles (UCLA) Fielding School of Public Health, University of California, Los Angeles, California, United States of America; 6 Department of Family Medicine, David Geffen School of Medicine at UCLA, Los Angeles, California, United States of America; 7 UCLA Clinical and Translational Science Institute, Population Health Program, Los Angeles, California, United States of America; 8 Department of Health Policy and Management, UCLA Fielding School of Public Health, Los Angeles, California, United States of America; 9 Associate Dean for Clinical Affairs, University of Southern California Alfred E. Mann School of Pharmacy and Pharmaceutical Sciences, Los Angeles, California, United States of America; University of KwaZulu-Natal College of Health Sciences, SOUTH AFRICA

## Abstract

**Introduction:**

Community-based health interventions often demonstrate efficacy in clinical trial settings but fail to be implemented in the real-world. We sought to identify the key operational and contextual elements of the Los Angeles Barbershop Blood Pressure Study (LABBPS), an objectively successful community-based health intervention primed for real-world implementation. LABBPS was a cluster randomized control trial that paired the barbers of Black-owned barbershops with clinical pharmacists to manage uncontrolled hypertension in Black male patrons, demonstrating a substantial 21.6 mmHg reduction in systolic blood pressure. Despite this success, the LABBPS intervention has not expanded beyond the original clinical trial setting. The aim of this study was to determine the facilitating and limiting factors to expansion of the LABBPS intervention.

**Methods:**

We undertook a qualitative assessment of semi-structured interviews with study participants performed after trial completion. Interviews included a total of 31 participants including 20 (6%) of the 319 LABBPS program participants (“patrons”), 10 (19%) barbers, and one (50%) clinical pharmacist. The semi-structured interviews were focused on perceptions of the medical system, study intervention, and influence of social factors on health.

**Results:**

Several common themes emerged from thematic analysis of interview responses including: importance of care provided in a convenient and safe environment, individual responsibility for health and health-related behaviors, and engagement of trusted community members. In particular, patrons reported that receiving the intervention from their barber in a familiar environment positively influenced the formation of relationships with clinical pharmacists around shared efforts to improve medication adherence and healthy habits. All interviewee groups identified the trust diad, comprising the familiar environment and respected community member, as instrumental in increasing health-related behaviors to a degree not usually achieved by traditional healthcare providers.

**Discussion:**

In conclusion, participants of an objectively successful community-based intervention trial consistently identified key features that could facilitate wider implementation and efficacy: social trust relationships, soliciting insights of trust bearers, and consistent engagement in a familiar community setting. These findings can help to inform the design and operations of future community-based studies and programs aiming to achieve a broad and sustainable impact.

## Introduction

A large number of evidenced-based interventions to improve health behaviors, including community-based programs designed to bring interventions directly to individuals, have been developed and rigorously evaluated with respect to their ability to significantly impact various clinical outcomes [1]. A prime target for community-based interventions, hypertension (HTN) represents the leading modifiable cardiovascular risk factor and disproportionately affects several racial and ethnic minority populations, particularly non-Hispanic Black men [2–4]. To help address this gap in health equity, the Los Angeles Barbershop Blood Pressure Study (LABBPS) evaluated the effectiveness of a community-based HTN intervention in which trained clinical pharmacists diagnosed and managed HTN among non-Hispanic Black men in Black-owned barbershops [5]. In this cluster randomized control trial, 52 Black-owned barbershops in Los Angeles County were randomly assigned to an intervention arm in which barbers encouraged meetings with trained clinical pharmacists who provided HTN treatment in the barbershops or a control arm in which barbers provided educational pamphlets on HTN and encouraged physician appointments [5]. After 6 months, the intervention group had a statistically significant reduction in systolic BP of 21.6 mmHg compared to the control group, with results sustained at 12 months [5,6].

Despite the resounding success of the LABBPS intervention, barbershop based HTN management has not moved widely outside of the clinical trial setting [7]. Unfortunately, this lack of translation of a positive, cost-effective intervention into practice is not uncommon in clinical research. To overcome these barriers and effectively translate community-based research interventions into a real-world setting, further adaptation of interventions is often required to further minimize organizational costs while maintaining efficacy. Thus, an in depth understanding of the most salient components of the LABBPS research protocol from the perspective of participants is required to expand the LABBPS intervention outside the context of a clinical trial. There is abundant quantitative research on the health inequities of non-Hispanic Black men, but far less on the nuanced aspects of their individual beliefs and behaviors, as well as the social context that affects their views and openness to medical intervention. The aim of this qualitative study was to determine the facilitating and limiting factors to expansion of the LABBPS intervention, and addresses the gap in knowledge through standardized analysis of semi-structured interviews with LABBPS barbers, shop patrons, and a clinical pharmacist responsible for facilitating the program.

## Methods

We conducted key informant interviews with 10 barbers, 20 patrons and 1 clinical pharmacist (n = 31) who were a part of the LABBPS cluster randomized control trial. The current study consists of a qualitative analysis of semi-structured interview guides/scripts tailored to each interviewee’s role and containing multiple layers of questions (primary, secondary, probes) which were developed by a multidisciplinary team of researchers, clinicians, and public health experts with experience in evaluating public health program scalability (**S1 File**). Interview domains included participants’ experience with the medical system and the LABBPS intervention, the individual factors that influenced their participation and HTN management, and the social factors that influenced their decision to participate in the study and focus on HTN control. The interviews were completed between July and August 2020; each interview lasted approximately 20–30 minutes. Interviewees were not paid for their participation.

Barbers and shop patrons from the intervention arm of the LABBPS were contacted by a member of the study staff via phone or text message and invited to participate via convenience sampling. The predetermined number of participants was 20 (6% of the original 319) and the predetermined number of barbers was 10 (19% of the original 53). These sample sizes were deemed to be of an adequate size to achieve data saturation. Those who expressed interest in participating were enrolled sequentially until the prespecified allotment of participants and barbers were reached. No approached study patrons or barbers declined participation. Based on the inclusion criteria of the LABBPS, all patrons were non-Hispanic Black men who regularly visited the same barbershop in their neighborhood [5,6]. Of the two clinical pharmacists, the one with the highest patron contact was selected, invited, and agreed to participate.

Due to the ongoing COVID-19 pandemic during the study period, interviews were conducted by a trained study staff member by phone or utilizing a web-based video platform (virtual meeting). The interviewer (M.R.) did not have any prior contact with the barbers or patrons who participated in the interviews. All patrons were English-speaking, and the interviews were conducted in English. All interviews were recorded (audio only) and subsequently transcribed using standard protocols. Data analysts knew the interviewee’s role in the LABBPS but had no other identifying information.

Analyses of the interview transcripts were performed in accordance with the consolidated criteria for reporting qualitative research (COREQ) checklist (**S2 File**) [8]. The study design was based in grounded theory, using thematic analysis and taking an inductive approach. This process included a preparatory phase during which two members of the study team (N.K. and R.F.), not involved in the initial LABBPS intervention, read interview transcripts. They independently compiled a list of themes derived from the transcripts and established a coding process to capture subject behavior, experiences, and attitudes, linking phrases of text to each identified theme. Themes were then modified and trimmed during a second independent transcript review. Next, independently developed themes were reconciled between N.K. and R.F., in conjunction with a third study team member (N.C.B.), again not involved in the original LABBPS intervention. All themes were then shared among study team members for input to reduce overlap and to promote adherence to the use of the previously agreed upon content analysis framework.

All study protocols/procedures and materials were reviewed and approved by the Cedars-Sinai Medical Center (Pro00035347) and Los Angeles County Department of Public Health Institutional Review Boards. Written informed consent was obtained from all participants, including consent to record their interview. Study enrollment occurred from February 17, 2015 through June 20, 2017. Due to the sensitive nature of the data collected for this study, requests to access the dataset from qualified researchers trained in human subject confidentiality protocols may be sent to Cedars-Sinai Medical Center at biodatacore@cshs.org. Data storage is managed by the Cedars-Sinai Research Compliance Office and data will be maintained in a manner and for a period of time in compliance with institutional requirements.

## Results

From the 52 barbershops, 319 barbershop patrons, and 2 clinical pharmacists who participated in the LABBPS, we were able to achieve target enrollment of each of the three groups as planned: 10 (19.2%) barbers from different shops, 20 (6.3%) shop patrons, and 1 (50%) pharmacist. In total, 31 key informant interviews were conducted. Nine out of the 10 barbers we spoke with had been barbers for greater than 20 years, 6 of whom had worked at a single shop over that period. The aim of this study was to determine the factors making the expansion of the LABBPS intervention easier or more difficult, and to that end, the three notable themes on individual and social factors that influenced participation, experience, and clinical outcomes of those who were involved in the intervention, are described below.

### Theme 1: The intervention facilitated convenient, safe experiences for participants

Most barbers, shop patrons, and the pharmacist described their experiences with the intervention program as novel, convenient, and comforting (**Table 1**). For example, one barber said that convenience and comfort played essential roles in convincing their clients to enroll in the LABBPS since many of them will “come to the barbershop, but they won’t go to the doctor” (Barber 3). Also, community-based provision of the intervention [5,6] in the barbershop demonstrated to patrons that they matter, that there is “respect for a community that they’re trying to reach” (Barber 3). Another barber said that getting an endorsement from a barber was a great way of putting Black men—a group with historical mistreatment in medical settings [9]—at ease. “Once I explained to them what it was, then it was perceived in a much better light” (Barber 1). This sentiment was echoed by other barbers who believed that their long tenure in the neighborhood was an important component of the trust that was generated. One barber noted, “Well, the people in the community already know who I am and what I’m about. So [they] know that I’m here to help instead of hurt” (Barber 6). Eight of the 10 barbers commented that the study had culturally appropriate tailoring, by explicitly reaching out to Black men in barbershops. Eight of 10 also said that the use of free haircuts as incentives to recruit participants was effective throughout the trial, and a majority (6/10) reported they used unassertive language with their patrons to avoid alienating them from the intervention. A majority (6/10) also said that compensating barbers for their work was an important element of recruiting and retaining barbers in the study.

**Table 1:** Interviewee experiences with the LABBPS intervention. Identified viewpoints from thematically analyzed semi-structured interviews barbers, shop patrons, and a pharmacist who participated in the research trial, supported by representative quotations.

Viewpoint(s)	Quotes
**Barbers’ perspective**	
Culturally tailored, with barbershop at center.	“At first, any medical [intervention] amongst Black people was [going to need an explanation before] it could proceed. Well, once I explained to them what it was, then it was perceived in a much better light.” (Barber 1)
Subtle encouragement from barber	“It was, ‘Hey, go ahead and just try it, just go ahead and do it.’ … That was the biggest thing.” (Barber 3)
Barbers with 20+ year ties to community.	“Well, the people in the community already know who I am and what I’m about. So [they] know that I’m here to help instead of hurt.” (Barber 6)
Financial incentive for barber.	“It’s time consuming. It will get more barbers involved… if they had an incentive.” (Barber 8)
**Shop patrons’ perspective**	
Widespread goodwill from patrons and barbers.	“You know there’s so many other things out there that we don’t have opportunities in the world [to do]. So this was a blessing. And I was able to take advantage of it because it was in the barbershop.” (Patron 17)
Showing results motivated patrons.	“Seeing that my blood pressure was going down each week, it really had me excited and motivated to continue on with this.” (Patron 7)
Open to video chat.	“Well, actually I think the video chat would be cool.” (Patron 2)
Financial incentive for patron.	“The monetary part was very good. It was a good incentive, but the benefit it was to keep me on point, [and] know that I need to stay in shape.” (Patron 6)
**Collectively, on the issues of convenience, comfort, and longitudinal (follow-up) care**	
Convenience and comfort. Meeting people where they are.	"People were more open and willing to share things [in the barbershop] that they wouldn’t typically share in a medical setting… As providers we’re going to have to get comfortable with leaving our hospitals to provide care in places that are convenient to our patients.” (Pharmacist 1)
Consistent comprehensive longitudinal care.	“[The pharmacist] was very instrumental in saying, we’re going to try this. This is for your own good. Please keep your appointments and 26-28keep tracks. She was kind of urging me to help myself.” (Patron 11)

Shop patron interviewees reported similar views, with 19 of 20 stating that convenience and comfort represented major factors in their participation. Eleven indicated they were excited and motivated by seeing improvements in their blood pressure, as well as regular follow-up from the pharmacist during the study. Collectively, barbers, shop patrons, and the pharmacist interviewed all reported that meeting patrons in a familiar location and understanding individual care needs, as well as the intervention’s longitudinal design (compared with one time screening programs), were appealing features that enhanced participant retention in the program.

### Theme 2: Trusted community members helped participants improve perceived individual responsibility over health-related behaviors

Members from all 3 groups indicated that engagement from trusted individuals contributed to improved self-efficacy of participants as it related to controlling their blood pressure. The components that were most frequently identified as improving participant agency over their hypertension were improved knowledge of their blood pressure and blood pressure management, understanding of the contribution of health-related behaviors to blood pressure, and beliefs around individual responsibility for maintaining health (**Table 2**).

**Table 2:** Individual factors that influenced LABBPS intervention participation and HTN management. Identified knowledge, behavior, and belief-based themes that contributed to intervention success from analyzed semi-structured interviews barbers, shop patrons, and a pharmacist who participated in the research trial, supported by representative quotations.

At the Individual Level	Quotes
**Knowledge**	
Unfamiliar with medication regimen.	“I forgot all those names. It was only one prescription and I forgot that, right? Yeah. It was a water pill…” (Patron 5)
Aware of healthy BP habits.	“Medication and diet and exercise. That was the plan before I started to study." (Patron 16)
Barbers learning HTN patterns.	“Being involved in the study definitely enlightened [me] with respect to what exactly your number should be, [and] what we were looking for in someone to bring the numbers down.” (Barber 1)
Barbers educating shop patrons.	“You still have to make lifestyle changes…The problem with this particular disease is that people don’t know they have it. They don’t feel any different.” (Barber 1)
**Behaviors**	
Patron used strategies to take medications.	“Yeah, just have to get in a mood and just remember to take your medicine prior to going to bed. And if you have to take it in the morning, when you wake up, you just have to do little things to combat now for the same thing. (Patron 15)
Patron had increased self-efficacy about HTN management after the study had ended.	“The smoking and the drinking and the cigars—you have to really be frank and open up and realize that these things, although they might be kind of pleasurable, are not really being helpful to you. It just made you aware that this is something that you need to address it if you’re really serious about taking care of yourself.” (Patron 18)
**Beliefs**	
Health depends on individual will power.	“I had to do my job, you know. I have to comply and make sure I take my meds and not miss a day.” (Patron 3)
Skepticism of peers’ attention to health	“A lot of us don’t really care. I didn’t care at first.” (Patron 5)
Appreciated personalized care.	“I do remember [the pharmacist] greeting me and welcome me back [to] prep for the study, asking me questions. [She asks what’s happened] between the last visit and the new visit, things that I’ve been doing know, [if] I haven’t been taking the pills so regularly. Like the traditional follow up questions.” (Patron 17)

Barbers expressed that individual patron behavior was paramount in improving HTN control and obtaining value from participation in the program. All expressed the importance of individual responsibility in monitoring and controlling chronic health conditions, particularly the role of individual health behaviors and habits in determining long term health. All but one barber said that the study intervention helped patrons improve health related behaviors. The importance of individual behavior was echoed by all 20 patrons who expressed beliefs that their health was their individual responsibility; 19 of 20 patrons reported that participating in the intervention improved their self-efficacy (individual health agency). Eighteen of the 20 said they were aware of healthy behaviors or habits that could lower their blood pressure before participating in the study, with 16 stating without prompting that their peers were not as attentive to their health as needed. Finally, 13 of the 20 shop patrons said that participating in the study intervention taught them individual strategies that they can use to adhere to their medication regimens.

### Theme 3: Trusted community members promoted intervention participation and retention

All interviewed barbers and patrons emphasized the importance of relationships and trust—specifically the barbershop serving as a trusted neighborhood hub and wanting to stay healthy for one’s family—in influencing their participation and care-seeking behaviors to manage blood pressure control and improve their health (**Table 3**). For example, every barber viewed participation in the intervention as a way of helping their community, as well as educating their patrons about hypertension. Of the ten interviewed barbers, 8 observed that the LABBPS relied on them to refer patrons to the intervention, while 6 noted that the blood pressure checks became a normal part of their barbershop routine.

**Table 3:** Social factors that influenced LABBPS intervention participation and HTN management. Identified relationship, institutional, and trust-based themes that contributed to intervention success from analyzed semi-structured interviews barbers, shop patrons, and a pharmacist who participated in the research trial, supported by representative quotations.

Social Condition/Element	Quotes
**Relationship building**	
Discussed care with family and friends.	“I’m giving away information to my family and my friends and my community, letting them know that you have to get your blood pressure checked. If you don’t, you won’t know, because this is a silent killer.” (Patron 7)
Discussed care with barber.	“The barber, he’s had pressure problems, you know, so… we both look forward to having a test because we would… compare. Pressure up pressure down? It’s like a competition.” (Patron 19)
Valued personal relationship with the pharmacist.	“She was very concerned and caring. It just made me comfortable and to want to [do] the most about this study. [It was the] availability and the means to have somebody to be there when you need them.” (Patron 8)
**Institutional environment**	
Skepticism about medicine.	"The actual doctor, he doesn’t look into your entire lifestyle and then say, ’Okay, I think should be on this or that.’ He just [gives you] medication.” (Patron 9)
Blood pressure measurement normalized in barbershop.	“When I looked around, I’ve been knowing the barbers in there for a while now and all [of them] were [working in the LABBPS trial]. So I said it wouldn’t hurt me to do it too." (Patron 12)
**Forming trust**	
Believe pharmacist cared about them.	“It felt like they cared. I felt like they really wanted to see me get my blood pressure down and change my diet… They wanted me to do better.” (Patron 5)
Barbers’ referral of patrons into study.	“[It was] different when people would talk about [how] the study was here. [We] then told them that if their blood pressure was kind of high and then if they were having trouble with it, they could have their blood pressure checked here… And that’s how they got into it.” (Barber 9)

All 20 patrons reported that they discuss their health with friends and family, while 18 said they discussed their care with their barber. Seventeen said it was important to them that the intervention pharmacist “cared” about them and personalized their care regimen. Importantly, while a majority (11/20) expressed unsolicited skepticism about the medical establishment in general, 16 reported they valued their personal relationship with the program pharmacist.

## Discussion

The aim of this thematic analysis was to determine novel and nuanced insights regarding the aspects critical to the success and scaling of a landmark community-based pharmacist-guided HTN treatment program for non-Hispanic Black men delivered in Black-owned barbershops [5,6]. Based on interview transcripts with 31 trial participants, three primary themes were identified across participant groups. These included i) provision of care in a convenient and safe environment; ii) individual responsibility for health and health related behaviors, and; iii) engagement of trusted community members. These findings demonstrate the importance of social and contextual factors as essential elements of community-based participatory research interventions [10], provide guidance to efforts aimed at ‘iteration-to-implementation’ of protocols, and are generalizable to community-based interventions targeting other disease states.

The LABBPS intervention represented a novel and exceedingly efficacious BP treatment program, reducing systolic BP by over 20 mmHg in a cluster randomized control trial [5,6]. Follow up cost-effectiveness analyses also indicate the potential for the program to be financially viable, finding an estimated cost of $17,162 per quality adjusted life year gained, well below current accepted cost thresholds [11]. Furthermore, projections indicate that broad scale adoption of the LABBPS model could prevent as many as 8,600 major adverse cardiovascular events annually [12]. Despite these compelling facts, similar to other community-based health studies that bring interventions directly to individuals, particularly those developed for medically underserved populations [13–17], the LABBPS intervention has not widely expanded to non-study locations, even at a local level. Expansion of community-based interventions often face significant hurdles including lack of sustainable funding [18] and limited organizational capacity [10]; overcoming these factors often requires significant changes to core programmatic components vital to an intervention’s success as part of attempts to minimize the program’s organizational and financial costs. An illustrative example is the Merck Community Asthma Network (MCAN), a community participatory research intervention aimed at reducing asthma complications in school age children in the Los Angeles Unified School District. The MCAN program began with identifying 12 evidenced-based interventions found to be efficacious in reducing asthma complications in randomized control trials and meta-analyses. However, without an understanding of operational factors required for uptake, the program failed to spread throughout schools [19–21].

Our results highlight the importance of the social context in which a program is implemented on participants’ views and openness to medical intervention, a crucial dynamic to understand given the manner in which many populations, particularly non-Hispanic Black patients, have been traditionally underserved by the medical establishment [22]. We found that LABBPS’ community-participatory research design [5,6] was a large contributor to its success, with barbers and pharmacists performing central roles in designing the intervention, and subsequently recruiting, retaining, and supporting patrons who enrolled.

Importantly, the relationship between the 3 major identified themes was not hierarchical, but rather relational in that each theme was valid independent of the others, however, all 3 were considered requisite to achieve optimal intervention outcomes. For example, while prior research has identified the importance of a comforting environment as a facilitator of effective care delivery [23,24], this was augmented in the LABBPS intervention by the pairing of this environment with a trusted community member (the barber). Similarly, while the importance of intervention salience is well established as a key driver for program uptake, this was enhanced in LABBPS through community-based discussions with barbers, family members, and other members of the non-Hispanic Black male community.

The importance of trust between patient and provider, as well as patient and community members are evidenced by the number of patrons who reported discussing their personal health with their family, barber, and the program pharmacist. This trust has often proven difficult for researchers to quantify and care providers to attain when treating non-Hispanic Black men [25,26]. LABBPS patrons reported, and interviewed barbers and pharmacists echoed, that the intervention team reinforced this trust by asking participants about their health goals and working with them to achieve them, rather than treating participants merely as a means to collecting health metrics. In the intervention, barbers and pharmacists made concerted efforts to better understand the individual and societal factors effecting each patron such as prior attempts (and failures) to control blood pressure, scheduling constraints related to dosing regimen, and factors that would motivate that individual to maintain adherence to their antihypertensive regimen [5,6]. This process enhanced participant trust in the intervention and facilitated tailoring HTN management and self-care strategies for each individual. With the goals of patron, barber, and pharmacist aligned, treatment adherence was achieved at a much higher level than typically expected. In this regard, the LABBPS stands in contrast to past community-participatory health interventions including the LABBPS’ predecessor study, BARBER-1, in which barbers screened patrons for high blood pressure, but referred them to their primary care provider for management, rather than providing in-shop treatment [27]. BARBER-1 demonstrated a much lower and statistically insignificant reduction in systolic blood pressure (-2.5 mmHg, P = 0.08) which was attributed in part to insufficient trust and comfort of participants with the medical system.

While patrons expressed a general distrust of the medical establishment, they overwhelmingly reported to have valued their relationship with the study pharmacists. This was attributed to several factors including direct contact with the pharmacist over the phone, presence of the pharmacist in their shop, and the ability to meet with the pharmacist is public community locations outside of the shops in between haircuts. Built trust with pharmacists, paired with existing trust with their barbers, resulted in improved receptivity of patrons to education on the importance of HTN management and health related behaviors to their health, and enhanced salience of the intervention in achieving these goals. Importantly, the LABBPS intervention was designed using principals of community participatory research, with insight from non-Hispanic Black patients and community leaders [5,6] (such as barbers and patrons) enabling the creation of a program that leveraged both existing relationships and fostered new trusting connections, resulting in an intervention more acceptable to the target population than would have been created without these insights.

Several limitations of this study merit consideration. First, the interviews did not involve participants of standard care (control) group, thus, leaving some gaps in information about the experiences of those who did not receive barbershop based HTN management from a pharmacist. However, the goal of the present analysis was to obtain insights on the LABBPS intervention, which control group participants did not experience. Second, qualitative methods are frequently prone to social desirability bias as they relate to how interviewees may respond based on their perceived understanding of what the interviewer may expect. To address this possible bias, interviews for this study were conducted by an interviewer who had no prior contact with the interviewees. Third, the decision was made to interview a pre-determined number of study of barbers and barbershop patrons, rather than interviewing new participants until data saturation was reached. However, analysis of the transcripts suggests that data saturation was achieved within the pre-determined samples. Additionally, interviews were specific only to the intervention experiences, excluding non-intervention related experiences or other conditions. Finally, this analysis focused on a HTN-specific intervention targeting the non-Hispanic Black male community, which may limit its generalizability. Importantly, however, the thematic analysis was tailored to generate elements that were broadly applicable to community-based interventions and may be extrapolated to other disease states and populations.

## Conclusion

Qualitative analysis of semi-structured interviews from participants in a successful community-based blood pressure reduction intervention [5,6] found that programmatic success was facilitated by provision of care in a familiar environment, encouragement from trusted community members, and the creation of a sense of personal responsibility among study participants. These factors emerged as necessary to success when scaling and expanding this intervention outside the confines of a clinical trial and may inform future efforts to streamline other community-based health interventions for broader implementation.

## Supporting information

S1 File Semi-structured interview questions. (PDF)

S2 File COREQ 32-item checklist. (PDF)
