# Supplementary material for: A qualitative analysis of post-hoc interviews with multilevel participants of a randomized controlled trial of a community-based intervention
Source: PLoS One. 2024 May 9;19(5):e0303075. doi: 10.1371/journal.pone.0303075 (PMC11081230; doi:10.1371/journal.pone.0303075)
Supplement: S1 File — (PDF) [file pone.0303075.s001.pdf]

Barber Semi-Structured Interview

Thank you so much for participating. I'm going to ask you a little bit about you and then will ask you a few questions about what you thought of the study. Let me know if you have questions at any time during this process.

1) Can you tell me a little bit about you? How long have you been a barber? How long have you been working at this barbershop?

2) How many clients do you see on a normal day?

3) How long does a haircut or other service normally take?

4) How many days a week do you work at the barbershop?

5) How long have you been seeing your clients? Do your clients tend to stay for many years?

Attitudes and Beliefs about Blood Pressure

1) *[Perceived Susceptibility]* Can you tell me about your health history? What are your biggest health concerns?
2) *[Perceived Susceptibility/Barriers]* What is your understanding of how blood pressure affects your health?
    a) Is this different than your understanding of blood pressure before the Barbershop Study? If so, how?
    b) Do you think it is difficult or easy for people to lower their blood pressure?
    c) Do you find it challenging to control your blood pressure?
    d) What do you do, if anything, to control your blood pressure?

Study Intervention

1) What were your initial thoughts when you were asked to participate in the Barbershop Study?
2) Were there any reasons that made you more likely to participate?
    a) Less likely to participate? If so, why were you willing to participate anyway?
3) What did you think about the study overall?
4) What did you like the most about the study?
5) What were the biggest challenges you had in the study?
6) What sorts of conversations did you have with your clients about blood pressure?

a) PROMPT: Did your clients tell you about why it was easy or hard for them to control their blood pressure?

7) When you were checking blood pressure for your clients, how many clients had normal blood pressure? (All, most, some, none)
   a) How many had high blood pressure?
   b) How many of those did you introduce to the Barbershop Study?
      i) How many of those were interested in participating?
      ii) What were reasons for not discussing the Barbershop Study with some clients?
   c) Did you screen every client? What were reasons that you didn't screen some clients?
8) For clients who declined participation, what reason, if any, did they give for not wanting to participate?
9) What was the general perception of your clients of the Barbershop Study?
   a) Were there any surprising reactions to the study? If so, in what way?

Perceptions of Study

1) How did you feel about having a way to check blood pressure and blood tests in your barbershop?

2) What was it like to check blood pressure for clients? Do you have any memorable moments around checking blood pressure?

3) Did your participation in the Barbershop Study change your interaction with any of your clients?

   a) If so, how? If not, why not?

4) Do you feel that the Barbershop Study was distracting from your daily work routine and if so, what elements were most distracting?

5) Do you feel the benefit of the study to your business outweighed the negatives?

6) Do you feel re-imbursement of some sort should go the barbers in similar programs and if so, why?

7) If you could go back in time, would you participate in the Barbershop Study again? Why or why not?

8) What else, if anything, do you think that the Barbershop Study staff should know about your participation or thoughts about this program?

   b) What about others in the medical community?

c) What about others in public health or government?

d) What about others in the community?

9) Do you feel that this program should be expanded to other barbershops? Why or why not?

Patron Semi-Structured Interview

Thank you so much for participating. I'm going to ask you a few questions about your health prior to participating in the study and then will ask you a few questions about what you thought of the study. Let me know if you have questions at any time during this process.

1) Can you tell me a little bit about you? How long have you lived in Los Angeles?
2) *[Perceived Susceptibility]* Can you tell me about your health history? What are your biggest health concerns?
3) *[Perceived Barriers]* Where do you normally go when you have a medical issue?
    a. Do you have a regular doctor?
    b. How often do you go see him or her?
    c. What do you think of your doctor?

Attitudes and Beliefs about Blood Pressure Control Prior to Study

3) *[Perceived Susceptibility]* Before starting the study, how important was it for you to lower your blood pressure?
4) *[Self-Efficacy]* Can you tell me what you did prior to the study to lower your blood pressure?
5) *[Perceived Susceptibility/Cues to Action]* Did your doctor or other health providers talk to you about reducing your blood pressure before you started the study? What were those conversations like?
    a) What did the doctor or health provider say?
    b) What did you think about these conversations?
6) *[Perceived Barriers/Benefits]* Prior to starting the study, were there things that made it harder or easier to control your blood pressure?
    a) Can you tell me about your blood pressure medications before the study?
    b) If you took medications prior to the study, were there things in your life that made it challenging to remember to take them?
    c) Oftentimes the cost of the medications can be challenging: was this an issue for you before the study?
7) *[Perceived Barriers/Benefits]* Do you have a significant other?
    a) Do you talk about your health with your significant other? What are those conversations like?
    b) Do you talk about your blood pressure?
    c) Are these conversations with your significant other helpful or not helpful?

d) Do you find that it helps to talk to family and friends about your blood pressure? If so, why?
e) Did you talk about the study?

Study Intervention

Attitudes and Beliefs about Study

1) What were your initial thoughts when you were asked to participate in the Barbershop Study?
   a) What motivated you to participate? What got you the most excited about the study?
2) *[Self-Efficacy]* Now that you have finished the study, what do you think about controlling your blood pressure?
3) *[Perceived Barriers]* What are the things in your life that make it the most difficult for you to keep your blood pressure down now, after the study has ended?
4) *[Perceived Benefit]* What did you like the most about the study?
5) *[Perceived Benefit]* What do you think was the biggest benefit of the study, if any?

Study Activities

1) Can you describe in your own words what you thought the purpose of the Barbershop study was?
   a) *If the participant did not describe the intervention, ask:* Describe in your own words the activities that the Barbershop Study staff did to examine this.
2) *The goal of the Barbershop Study was to see if a Clinical Pharmacist driven program in barbershops could decrease blood pressure in African American men with high blood pressure. To do this, blood pressure and lab tests were monitored in 52 local barbershops and Clinical Pharmacists.* What do you consider the most important aspect of what the Barbershop Study did? *Follow up question either a or b, depending on if the participant's blood pressure decreased during the study.*
   a) *[Self-Efficacy]* If your blood pressure went down, do you think there were specific activities or recommendations that were part of the study that were helpful?
      i) If not, what aspect, if any, do you feel was most important in reducing blood pressure?
      ii) What aspect of the Barbershop Study was least helpful in reducing blood pressure?
   b) If your blood pressure didn't go down, why do you think this was the case?
      i) *[Perceived Barriers]* What else was going on in your life during the Barbershop study?
   c) Would you add, change or remove any of the aspects of the barbershop study?
      i) If so, why?
3) How did you feel about having a way to check blood pressure and blood tests in your barbershop?

4) Did your participation in the Barbershop Study change your interaction with your barber?
   a) If so, how? If not, why not?
   b) What sorts of conversations did you have about the study?
5) Did you speak with your "regular" doctor during the study?
   a) If so, did you talk about your blood pressure?
      i) What did they say about the changes to your blood pressure and medications, if anything?

Interactions with Pharmacist

1) Can you describe your interactions with the Clinical Pharmacist?
   a) Can you tell me about your conversations with the pharmacist? What did you talk about? What did you find most helpful?
   b) How many times did you meet in person?
   c) Text?
   d) Speak on the phone?
   e) How important was it for you to have a personal connection with the pharmacist?
   f) Do you think this program would work with video-chat only (no in-person visits)?

Medication Adherence

1. Can you tell me about your blood pressure medications during the study?
   a) What recommendations did the pharmacists give you about your medications?
   b) Which were the most helpful? Which were the least helpful?
   c) Were there things in your life that made it challenging to remember to take them?
2. Tell me about your blood pressure medications today?
   a) What are you taking? Have your medications changed since the study?
      i) If so, why?
   b) Are there things in your life that make it challenging to remember to take them?
   c) What helps you remember to take your medications?

Perceptions of Study

1. If you could go back in time, would you participate in the Barbershop Study again? Why or why not?
2. What else, if anything, do you think that the Barbershop Study staff should know about your participation or thoughts about this program?

a) What about others in the medical community?

b) What about others in public health or government?

c) What about others in the community?

3. Do you feel that this program should be expanded to other barbershops? Why or why not?

Pharmacist Semi-Structured Interview

Thank you so much for participating. I'm going to ask you a few questions about you, then we will talk about your interactions with the study participants and then will ask you a few questions about what you thought of the study. Let me know if you have questions at any time during this process.

1) What do you like about being a pharmacist?

2) How long have you been a pharmacist?

3) What are the most challenging parts of being a pharmacist?

Overall Impression of the Study

1) What were your overall impressions of the study?

2) What were the biggest challenges in implementing the study?

3) What did you like the most about the study?

4) Was there anything surprising about the study?

5) What do you feel was the most important aspect of the intervention? If different, please indicate important aspects in terms of both for reducing blood pressure and for the overall success of the study.

f) How about least important?

Interactions with Study Participants

1) Can you tell me a little about your interactions with the study participants?

2) Were there interactions that were very easy or very challenging? Can you tell me more?

3) Were there any times you felt frustrated during these interactions?

4) Did participants talk about their thoughts about the study to you? If so, what were those conversations like?

5) How many times did you meet with participants in person, on average?

a) Were there some participants with whom you interacted more or less? Why do you think that was? What accounted for the different levels of participation, in your opinion?

g) What about texting? What did participants think of texting? Were some more or less into it?
h) What about talking on the phone? What did participants think of talking on the phone? Were some more or less into it?
i) What did you think about the driving? How much time, on average, did you spend driving? Was this challenging for you?

Interactions with Other Healthcare Providers

1) Did you speak with of the participants' regular doctors or healthcare providers during the study?
    a. What were these conversations like?
    b. Was it challenging to get in touch with their regular providers? If so, why? If not, why not?

Perceptions of Study

1) If you could go back in time, would you participate in the Barbershop Study again? Why or why not?
2) What else, if anything, do you think that the Barbershop Study staff should know about your participation or thoughts about this program?
    a) What about others in the medical community?
    b) What about others in public health or government?
    c) What about others in the community?
3) Do you feel that this program should be expanded to other barbershops? Why or why not?
4) What do you see as barriers to expanding the study to other barbershops?
5) What do you see as factors that would make it easy to expand the study to other barbershops?
