# Supplementary material for: A qualitative analysis of post-hoc interviews with multilevel participants of a randomized controlled trial of a community-based intervention
Source: PLoS One. 2024 May 9;19(5):e0303075. doi: 10.1371/journal.pone.0303075 (PMC11081230; doi:10.1371/journal.pone.0303075)
Supplement: S2 File — (PDF) [file pone.0303075.s002.pdf]

A Qualitative Analysis of Post-Hoc Interviews with Multilevel Participants of a Randomized Controlled Trial of a Community-Based Intervention

COREQ 32-item checklist

No. Item	Guide questions/description	Reported on Page #
**Domain 1: Research team and reflexivity**		
Personal Characteristics		
1. Inter viewer/facilitator	Which author/s conducted the interview or focus group?	Page 7 / paragraph 3
2. Credentials	What were the researcher's credentials? E.g. PhD, MD	Page 1 / paragraph 1
3. Occupation	What was their occupation at the time of the study?	**Research Fellow** (not reported in text)
4. Gender	Was the researcher male or female?	**Male**
5. Experience and training	What experience or training did the researcher have?	**Medical training**
Relationship with participants		
6. Relationship established	Was a relationship established prior to study commencement?	Page 7 / paragraph 2
7. Participant knowledge of the interviewer	What did the participants know about the researcher? e.g. personal goals, reasons for doing the research	Page 7 / paragraph 2
8. Interviewer characteristics	What characteristics were reported about the interviewer/facilitator? e.g. Bias, assumptions, reasons and interests in the research topic	Page 7 / paragraph 2
**Domain 2: study design**		
Theoretical framework		
9. Methodological orientation and Theory	What methodological orientation was stated to underpin the study? e.g. grounded theory, discourse analysis, ethnography, phenomenology, content analysis	Page 7 / paragraph 3
Participant selection		
10. Sampling	How were participants selected? e.g. purposive, convenience, consecutive, snowball	Page 7 / paragraph 1
11. Method of approach	How were participants approached? e.g. face-to-face, telephone, mail, email	Page 6 / paragraph 3

12. Sample size	How many participants were in the study?	Page 6 / paragraph 2
13. Non-participation	How many people refused to participate or dropped out? Reasons?	**None**
Setting		
14. Setting of data collection	Where was the data collected? e.g. home, clinic, workplace	Page 7 / paragraph 2
15. Presence of non-participants	Was anyone else present besides the participants and researchers?	**No**
16. Description of sample	What are the important characteristics of the sample? e.g. demographic data, date	Page 7 / paragraph 1
Data collection		
17. Interview guide	Were questions, prompts, guides provided by the authors? Was it pilot tested?	Page 6 / Paragraph 2 **It was not pilot tested**
18. Repeat interviews	Were repeat inter views carried out? If yes, how many?	**No**
19. Audio/visual recording	Did the research use audio or visual recording to collect the data?	Page 7 / paragraph 2
20. Field notes	Were field notes made during and/or after the inter view or focus group?	**No**
21. Duration	What was the duration of the interviews or focus group?	Page 6 / paragraph 2
22. Data saturation	Was data saturation discussed?	Page 7 / paragraph 1
23. Transcripts returned	Were transcripts returned to participants for comment and/or correction?	**No**
**Domain 3: analysis and findings**		
Data analysis		
24. Number of data coders	How many data coders coded the data?	Page 7 / paragraph 3
25. Description of the coding tree	Did authors provide a description of the coding tree?	Page 7 / paragraph 3<br>Page 8 / paragraph 1
26. Derivation of themes	Were themes identified in advance or derived from the data?	Page 7 / paragraph 3<br>Page 8 / paragraph 1
27. Software	What software, if applicable, was used to manage the data?	**Microsoft Word and Excel**
28. Participant checking	Did participants provide feedback on the findings?	**No**
Reporting		

29. Quotations presented	Were participant quotations presented to illustrate the themes/findings? Was each quotation identified? e.g. participant number	Page 20-22 Tables 1-3
30. Data and findings consistent	Was there consistency between the data presented and the findings?	Page 11 / paragraph 3 **Yes**
31. Clarity of major themes	Were major themes clearly presented in the findings?	Page 8 / paragraph 4 Page 10 / paragraph 1 Page 10 paragraph 3
32. Clarity of minor themes	Is there a description of diverse cases or discussion of minor themes?	Page 9 / paragraph 1 **Yes**

Developed from:
Tong A, Sainsbury P, Craig J. Consolidated criteria for reporting qualitative research (COREQ): a 32-item checklist for interviews and focus groups. *International Journal for Quality in Health Care*. 2007. Volume 19, Number 6: pp. 349 – 357
